# Emerging advances in identifying signal transmission molecules involved in the interaction between *Mycobacterium tuberculosis* and the host

**DOI:** 10.3389/fcimb.2022.956311

**Published:** 2022-07-25

**Authors:** Yue Wang, Qiyuan Shi, Qi Chen, Xuebin Zhou, Huiling Yuan, Xiwen Jia, Shuyuan Liu, Qin Li, Lijun Ge

**Affiliations:** ^1^ College of Life Science, Zhejiang Chinese Medical University, Hangzhou, China; ^2^ School of Pharmacy, Hangzhou Medical College, Hangzhou, China

**Keywords:** *Mycobacterium tuberculosis*, innate immunity, signaling pathway, immune escape, chronic infectious disease

## Abstract

Tuberculosis caused by *Mycobacterium tuberculosis* (MTB) is an ancient chronic infectious disease and is still the leading cause of death worldwide due to a single infectious disease. MTB can achieve immune escape by interacting with host cells through its special cell structure and secreting a variety of effector proteins. Innate immunity-related pattern recognition receptors (PPR receptors) play a key role in the regulation of signaling pathways.

In this review, we focus on the latest research progress on related signal transduction molecules in the interaction between MTB and the host. In addition, we provide new research ideas for the development of new anti-tuberculosis drug targets and lead compounds and provide an overview of information useful for approaching future tuberculosis host-oriented treatment research approaches and strategies, which has crucial scientific guiding significance and research value.

## Introduction

*Mycobacterium tuberculosis* (MTB), discovered in 1882 by the German bacteriologist Robert Koch, was proven to be the causative agent of human tuberculosis, which seriously endangers human health and public safety (Abrahams et al., 2018). After centuries of struggle, the disease has been gradually brought under control, but in recent years, it has become increasingly serious due to various factors. It is now one of the top ten causes of death worldwide and is the leading cause of death from infectious diseases. For example, according to the World Health Organization (WHO) 2020 World Tuberculosis Report, there were approximately 10 million new tuberculosis cases in 2019, of which approximately 3.3% of new tuberculosis cases and 17.7% of previously treated patients were MDR/RR-TB (multidrug-resistant tuberculosis/rifampicin-resistant tuberculosis). Three countries accounted for the largest proportion of drug-resistant tuberculosis, namely, India (27%), China (14%) and Russia (8%), with approximately 1.5 million deaths (Global tuberculosis report, 2020). Thus, the current situation of tuberculosis prevention and treatment remains very severe.

MTB is an intracellular bacterium, and the results of MTB infection largely depend on the host’s response to the invading pathogen and how the pathogen escapes from the host’s immune response. For this reason, elucidating the interaction between MTB and the host helps to explain the molecular mechanism involved in the infection of specific mycobacterium proteins (Brennan et al., 2007). The primary host cell targets of MTB are alveolar macrophages, which are the main effector cells for clearing MTB. After the MTB pattern recognition receptor expressed on the surface of macrophages binds to specific MTB surface-related molecules, macrophages can enact a variety of immune responses to eliminate MTB. Meanwhile, MTB utilizes a variety of mechanisms to escape the body’s immune killing to survive in its cells. As a typical intracellular bacterium, MTB has evolved a variety of immune escape strategies for long-term coexistence with the host (Barreteau et al., 2008). This review is followed by an introduction of the molecular mechanisms triggered by signal transmission molecules in the interaction between Mycobacterium and the host to provide new ideas for the study of tuberculosis infection and pathogenicity.

## Structural features of mycobacterium tuberculosis

**Figure 1:**
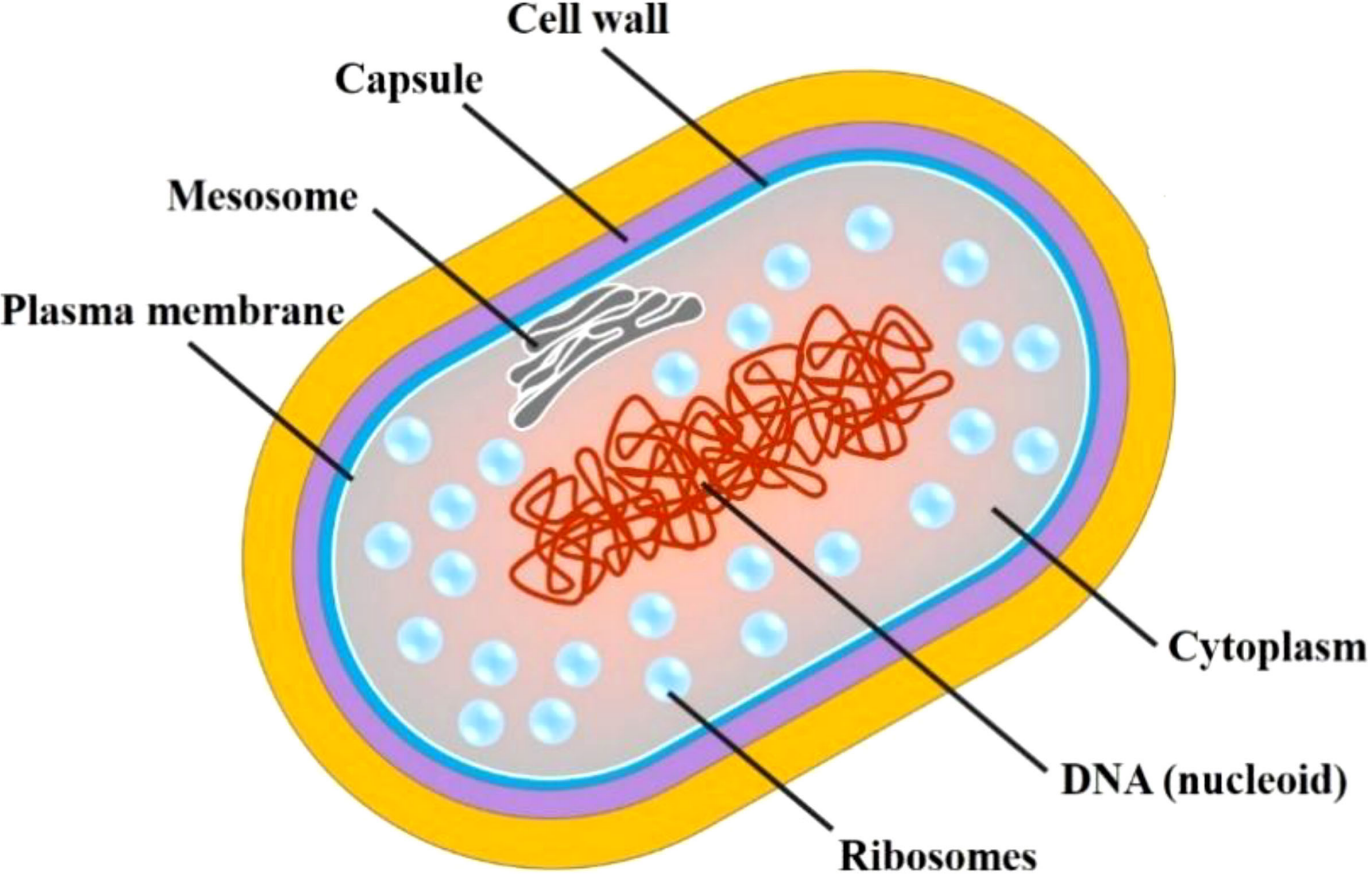
Cell structure of MTB.

MTB is an obligate, aerobic, slender, slightly curved bacillus, with a size of 1-4×0.4 μm, without flagellum, with fimbriae and microcapsules but without forming spores. The structural characteristics of MTB cells are summarized in **Figure 1**. MTB grows slowly mainly because the lipid content accounts for approximately 60% of the dry weight of the cell wall, which affects the absorption of nutrients (Marrakchi et al., 2014).

**Figure 2:**
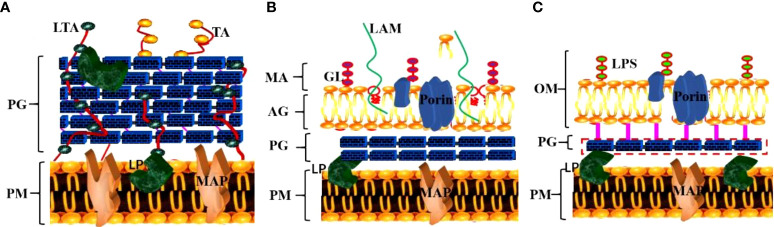
Specific structural components of the MTB cell wall. **(A)** The cell wall structure of gram-positive bacteria; **(B)** The cell wall structure of MTB; **(C)** The cell wall structure of gram-negative bacteria. (TA, teichoic acid; LTA, lipoteichoic acid; PG, peptidoglycan; PM, plasma membrane; LP, lipoprotein; MAP, membrane-associated protein; GL, glycolipid; LAM, lipoarabinomannan; MA, mycolic acid; AG, arabinogalactan; LPS, lipopolysaccharide; OM, outer membrane).

The unique cell wall components of MTB play a crucial role in immune escape and are also an important target of many anti-tuberculosis drugs (Kalscheuer et al., 2019). Compared with other pathogenic bacteria, the bacterial wall of MTB has neither lipoteichoic acid, teichoic acid (major wall components of most Gram-positive bacteria) nor lipopolysaccharide (major wall components of most Gram-negative bacteria) (**Figure 2**) (**Table 1**).

**Table 1:** Some immune escape molecules of MTB interacting with host cells.

Host cell PPR signal	MTB-related cell components and effector molecules			Target of host cell
	Cell wall Special components	The efflux pump system	Effector proteins	
TLRs	PG	ABC	PTPA	TRM27、GADD45A
NLRs	AG	MFS	MCE2E	eEFIA、MAPK
CLRs	MA	RND		ERK、JNK
CRs	TDM	SMR	MCE3E	ERK
MRs	MDP	MATE	ESAT-6	NLRP3

TLRs, Toll-like receptors; NLRs, Nod-like receptors; CLRs, C-type lectin receptors; CRs, Complement receptors; MRs, Mannose receptors;PG, Peptidoglycan; AG, Arabino galactan; MA, Mycolic acid; TDM, Trehalose-6,6’-dimycolate; MDP, Muramyl dipeptide; ABC, ATP-binding cassette; MFS, Major Facilitator Superfamily; RND, Resistance Nodulation Division; SMR, Small Multidrug Resistance; MATE, Multidrug and Toxic-Compound Extrusion; PtpA, Protein tyrosine phosphatase; MCE2E, MCE family proteins 2E; MCE3E, MCE family proteins 3E;ESAT-6, Early secretory antigentic target-6 protein; TRM27,Tripartite motif 27; GADD45A, Growth arrest and DNA damage inducible gene Gadd45; eEFIA, Eukaryotic translation elongation factor 1A; MAPK, Mitogen-activated protein kinase; ERK, Extracellular regulated protein kinases; JNK, c-Jun N-terminal kinase; NLRP3, NOD-like receptor thermal protein domain associated protein 3.

The cell wall of MTB is mainly composed of Peptidoglycan (PG), Arabino galactose (AG), Trehalose-6,6’-dimycolate (TDM), Mycolic acid (MA) and Muramyl dipeptide(MDP). The MTB cell membrane has a PG layer similar to that of gram-positive bacteria, which plays a key role in cell growth, communication, and stimulation of the host’s immune response (Kanji et al., 2019). The outer layer is the arabinan chain formed by highly branched AG, and the nonreducing end of the glycan chain is connected to the MA (Canezin et al., 2018) in the outer layer, which affects the permeability of dyes into MTB cells (Kleinnijenhuis et al., 2011). Thus, the outer layer of MTB, comprising predominantly lipids and carbohydrates, plays a crucial role in MTB survival and infection (Arcos et al., 2011).

In addition, many researchers have found that MTB can reduce the concentration of bacterial drugs through the efflux pump system, alleviate damage to itself, and induce drug resistance, which is another key mechanism of MTB immune escape (Richa et al., 2019). The efflux pump system of MTB is achieved by transporters located on the plasma membrane. There are currently five known MTB related transporter families, namely, ATP-binding cassette (ABC), Major Facilitator Superfamily (MFS), Resistance Nodulation Division (RND), Small Multidrug Resistance (SMR) and Multidrug and Toxic-Compound Extrusion (MATE) (Kallenius et al., 2016). Recent work indicated that THP-1 cells infected with MTB H37Rv and subsequently exposed to rifampicin for 72 hours expressed 10 efflux pump-related genes. Four genes in the ABC family and 1 gene in the MFS family were significantly increased at 0.0015 mg/L rifampicin (Benjawan et al., 2015). Therefore, the MTB surface proteoglycan and its unique Mycoplasma acid of its cell wall and the efflux pump on the plasma membrane are important mechanisms used to protect itself from injury and to promote immune escape.

## Mechanisms of interaction between MTB effector proteins and the host

The mechanism of MTB immune escape is related to its special cell wall structure as well immune damage caused by the toxicity of metabolites produced by MTB proliferation in host cells (Ji-Hae et al., 2021). MTB is an intracellular pathogen that secretes a variety of effector proteins into host cells, which in turn interfere with cell signaling pathways and biological functions and ultimately promote the survival of pathogens in host cells and lead to host cell lesions. For example, lipopolysaccharide (LPS) and lipoarabinomannan (LAM) in the cell wall of MTB can be recognized by TLR2/TLR1 or TLR2/TLR6 heterodimers on the membrane of host immune cells, which in turn activate the expression of NF-κB and cytokines, further leading to host cell injury (Wong, 2017).

**Figure 3:**
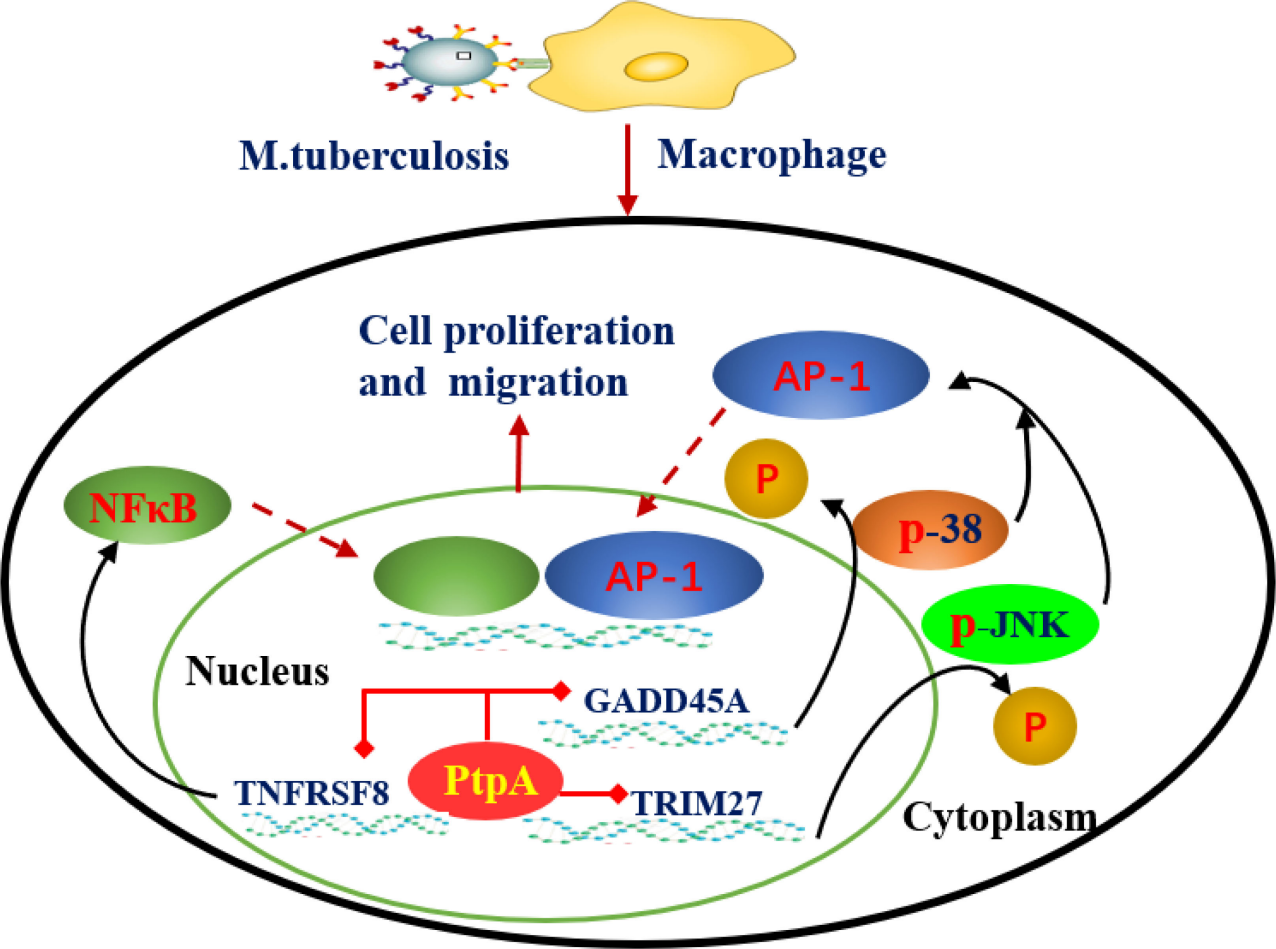
Mechanism by which MTB-PtpA enters the host cell nucleus to inhibit natural immune function. MTB PtpA is secreted into the host cell to bind ubiquitin molecules and is activated, and activated PtpA can inhibit the transcription of TNFRSF8 in the host nucleus, thus inhibiting the NF-κB signaling pathway. PtpA can also bind directly to the promoter region of the GADD45A or RNF187 gene and inhibit its transcription, dephosphorylating p-JNK and p-p38 to inhibit the activation of the JNK/p38 signaling pathway. (TNFRSF8, a member of the TNF-receptor superfamily; Trim27, belonging to E3 ubiquitin ligase family, can activate JNK/p38 signal pathway of host cells in a ubiquitin ligase activity dependent manner; GADD45A, a regulator of p38 MAPKs by inhibiting p38 phosphorylation and activity).

At present, recent studies have revealed the dynamic process and molecular mechanism of the interaction between MTB effector proteins, such as protein tyrosine phosphatase (PtpA), MCE family proteins (Mce2E and Mce3E) and the host cell. PtpA can be secreted into host cells to bind ubiquitin molecules and be activated by the latter, which in turn dephosphorylates host p-JNK and p-p38 and inhibits the activation of the JNK/p38 signaling pathway. For example, PtpA can antagonize the host interaction protein TRIM27 (a ubiquitin ligase) mediated JNK/p38 signal pathway by binding to the ring domain of TRIM27 protein (Jing et al., 2016). In addition, PtpA can also inhibit the activation of the NF-κB signaling pathway in a phosphatase activity-independent manner (Valérie et al., 2014; Mohd et al., 2021). Similar research results have shown that PtpA can regulate innate immune signaling pathways in the host cytoplasm as well as enter the host nucleus to regulate potential target genes, and PtpA can directly bind to the promoter region of the GADD45A gene and inhibit its transcription (Wang et al., 2017). The MTB genome contains four MCE (Mammaian cell entry) operons (Mce1-4), and the proteins encoded by them constitute a large class of MCE family. Previous studies have suggested that MCE family proteins play an important role in the entry, intracellular survival and pathogenesis of MTB, but its host regulatory function and mechanism are far from clear (Foot Perkowski et al., 2016; Singh et al., 2016). Recent studies have found that the Mce3E protein of MTB can specifically target the ERK signaling pathway of the host through its DEF motif (a MAPK binding motif), and ultimately promote the intracellular survival of mycobacteria. And MTB Mce2E operon contained a D motif (another MAPK binding motif) by further bioinformatics analysis. And the results showed that in macrophages, Mce3E specifically inhibited ERK signaling pathway through its DEF motif, while Mce2E simultaneously inhibited ERK and JNK signaling pathway through its D motif (Li et al., 2015). It is worth mentioning that Mce2E can also inhibit the K48 ubiquitination of the host cell proliferation promoting protein eEF1A1 in epithelial cells and increase its protein stability, thereby promoting the proliferation of human non-small cell lung cancer A549 cells derived from epithelial cells, while Mce3E does not have this function (Wei et al., 2019) (**Figure 3**). Therefore, these studies suggest that MTB can secrete a variety of effector proteins to co regulate some host signal pathways and functions, but the specificity and intensity of different effector proteins may be different. And the breakthrough of this research result will provide new ideas and specific targets for the future development of anti-MTB drugs from the perspective of the interaction between MTB effector proteins and host cells (Augenstreich and Briken, 2020).

In addition, it has been demonstrated that, when MTB enters the host lung tissue, the hydrolytic enzymes in alveolar lining fluid (ALF) can hydrolyze mannose and glucose of the MTB cell envelope molecules, thereby disrupting the structure of the MTB cell wall (Mika et al., 2013). In recent years, many researchers have suggested that this change significantly reduces the virulence factors mannose-capped lipoarabinomannan (Man-LAM) and trehalose-6,6’-dimycolate (TDM) on the surface of MTB (Tuladhar and Kanneganti, 2020). For example, MTB-secreted ESAT-6 protein can improve the permeability of phagosomes in host phagocytes and activate NLRP3 (Jain et al., 2020). Thus, after thousands of years of development, MTB has coevolved with humans. Its unique cell structure and metabolic effector proteins, in addition to the acquired drug resistance caused by gene mutations coexisting with the host under the action of drugs, have become primary reasons for the escape mechanism of MTB. MTB has become one of the most successful pathogens by changing its morphology, colonies, virulence, immunogenicity and drug resistance.

## Molecular mechanism of MTB escape from host PPR signal transduction

In recent years, the interaction between MTB and its host has become increasingly understood. However, at present, the molecular mechanism of the interaction between the secreted proteins of MTB and the proteins of the host cell is not completely clear, especially how MTB escapes from host pattern recognition receptor signaling (Allue-Guardia et al., 2021). Studies have demonstrated that MTB can change the immune function of host cells by secreting different effector proteins at different stages of infection. These results suggest that the immune escape of MTB is closely related to the host cell membrane surface pattern receptor (PPR) (Pathak and Das, 2020). For example, after entering the host, the cell wall components of MTB, such as phosphatidylinositol mannose (PIM), lipoarabinomannan (LAM), and some other outer membrane molecules, are recognized by macrophages through membrane surface pattern receptors. These receptors include Toll-like receptors (TLRs), Nod-like receptors (NLRs), C-type lectin receptors (CLRs) and complement receptors (CRs), mannose receptors (MRs), and Fc receptors of immunoglobulin (FcRs) (Cervantes et al., 2017). Most recognition and activation of the host immune system requires the above receptors to further activate downstream related signaling pathways and trigger host immune defense, including programmed cell death of different types, such as cell apoptosis and autophagy. Therefore, understanding the different innate recognition pathways of the host can help to reveal the pathogenic mechanisms of MTB and develop new control measures (Krakauer et al., 2019). Next, we will focus on the interaction between MTB effector proteins and host surface pattern receptors (PPRs), such as CLRs, NLRs, and TLRs, and the latest research on how MTB evades these receptors (**Table 1**).

### Molecular mechanism by which MTB escapes host TLR receptor signaling

Toll-like receptors (TLRs) are intrinsic pattern recognition receptors. The acquired immune response can be induced by the recognition of pathogen-associated molecular patterns (PAMPs) on the surface of specific microorganisms to promote the synthesis and release of cytokines and the maturation of antigen-presenting cells (Kawai et al., 2011). TLRs are a family of single membrane-spanning receptors that can be divided into three parts: the extracellular domain, cytoplasmic domain and transmembrane domain (Takeuchi et al., 2010). The extracellular domain of TLRs contains three extracellular domain proteins, namely, MD-1, MD-2 and RP105, which are used to recognize receptors and form receptor complexes with other coreceptors. The cytoplasmic domain of TLRs is highly homologous to that of IL-1R family members and is called the Toll-IL-1 receptor domain (TIR domain). There are 11 members of the TLR family. According to TLR protein localization and recognition substrates, they can be divided into two categories: plasma membrane-anchored TLRs and endosomal TLRs (Kumar et al., 2018). These are distinct molecular structures on microbes, and different sets of TLRs have been associated with the recognition of pathogens, such as the recognition of viruses by TLR3, TLR7, TLR8 and TLR9 (Kay et al., 2013; Liu et al., 2013; Hossain et al., 2013). TLRs mainly function through two signal transduction pathways, the myeloid differentiation factor 88 (MyD88)-dependent signaling pathway and the MyD88-independent signaling pathway, which induce the production of both proinflammatory cytokines and type I IFNs (Nguyen et al., 2020). These two distinct responses are mediated *via* the selective use of adaptor molecules recruited to the Toll/IL-1 receptor (TIR) domains of TLRs after ligand binding (Kawai et al., 2006). Thus, when TLRs are activated, they activate their downstream IL-1R-associated kinase (IRAK), tumor necrosis factor receptor (TNFR), and TNFR-associated factor 6 (TRAF-6), which further activate NF-κB and lead to immune and inflammatory responses (Liu et al., 2007). Many studies have confirmed that TLRs play a crucial role in TB infection (Doyle et al., 2006). For instance, among the TLR family, TLR2 can recognize lipoprotein, LPS, and CpG DNA at the cell wall of MTB and secreted membrane vesicles (Liu et al., 2018). In addition, it can activate the downstream NF-κB signaling pathway and stimulate the secretion of TNF-α to activate macrophages (Fiske et al., 2019). Meanwhile, it can stimulate the expression of vitamin D receptor and vitamin D-1-hydroxylase gene to induce the release of cathelicidins (Liu et al., 2021). Another study found that TLR2 could recognize the MTB LPQH (19 kDa lipoprotein) and lysosomal inhibiting LPRI and activate macrophages, which initiate innate immunity and activate T lymphocytes to participate in the clearance of MTB (Picard et al., 2003). TLR2 can form heterodimers with both TLR1 and TLR6. These heterodimers have been implicated in the recognition of mycobacterial cell wall glycolipids, including LAM, lipomannan (LM), mycobacterial glycoproteins (MGs), phosphatidylinositol mannitol (PIM), triacylated (TLR2/TLR1) and diacylated (TLR2/TLR6) lipoproteins (Bulut et al., 2005; Singh et al., 2019).

**Figure 4:**
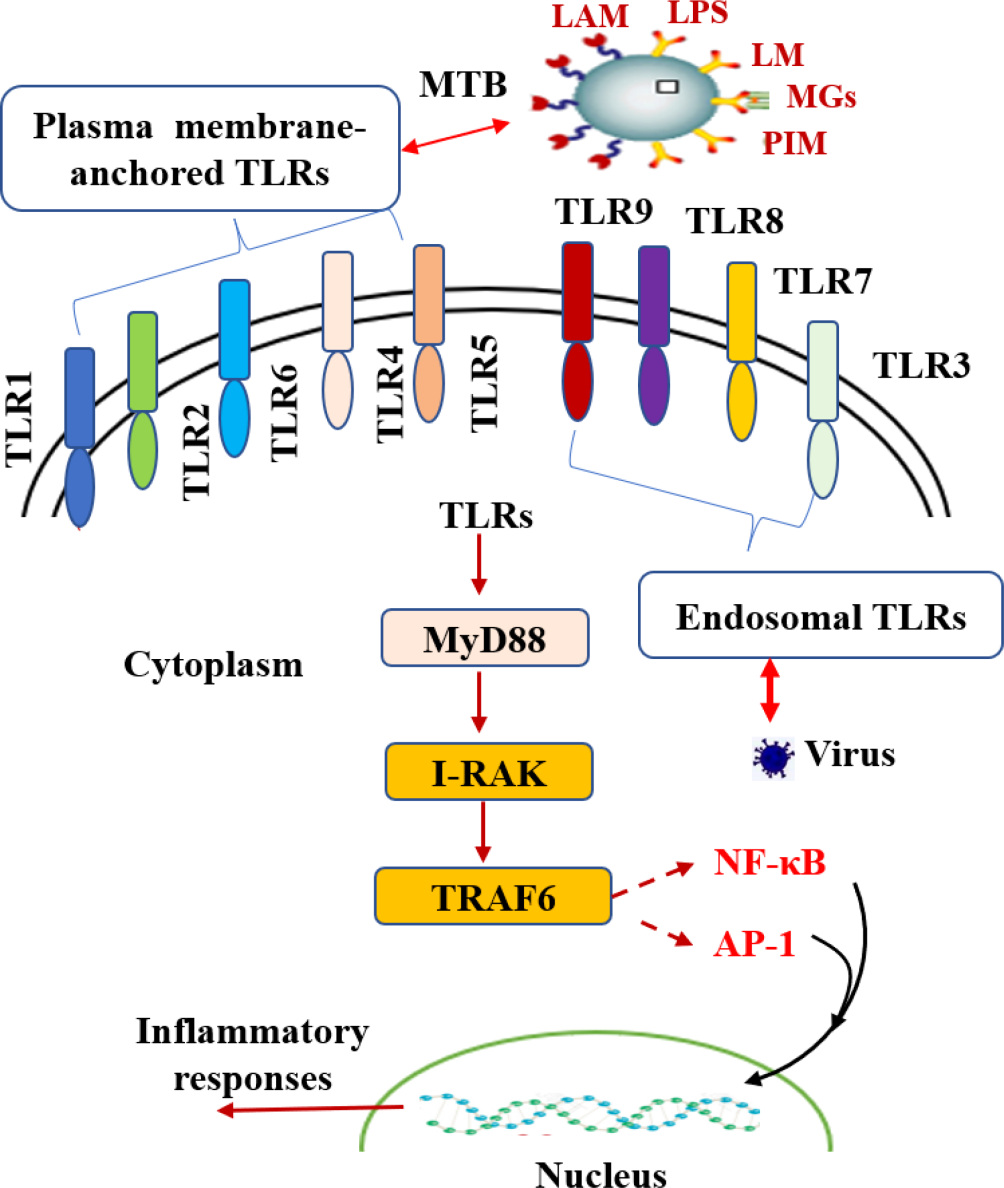
The interaction between TLRs and MTB infection. TLRs play a vital role in TB infection. Toll-like receptors are divided into plasma membrane-anchored TLRs and endosomal TLRs according to the localization of TLR proteins. As TLRs are activated, they activate downstream IRAK, TNFR and TRAF-6 through the MyD88-dependent signaling pathway, further activating NF-kB and AP-1 and producing a series of inflammatory reactions.

To date, studies agree that LAM, as a cell wall component of nonpathogenic mycobacterium, is a TLR2 activator with strong immunogenicity. LAM from pathogenic mycobacteria lacks immunogenicity (Drage et al., 2009). TLR2 is believed to be important in the initiation of the innate host defense against MTB (Kleinnijenhuis et al., 2009). In addition, IL-1β production is dependent upon TLR2 and TLR6 stimulation but not TLR4 or TLR9 (Yang et al., 2019). TLR2 polymorphisms affect host susceptibility to MTB. TLR2 ligands of MTB can change the host cell environment, which is conducive to bacterial retention (Konowich et al., 2017). MTB can affect the fate of infected cells by regulating the TLR signaling pathway. For example, LRP of MTB can inhibit the PI3K/Akt signaling pathway activated by TLR2 and inhibit the production of anti-inflammatory factors and macrophage antigen presentation. PtpA and Mce3E of MTB regulate the MAPK and NF-κB signaling pathways. Interestingly, it has been reported that GRP160 can also regulate the entry of MTB into macrophages through the MAPK/ERK signaling pathway, indicating that the GPCR and TLR signaling pathways may play a synergistic role (Mercedes Romero et al., 2014; Mehta et al., 2021). The interaction between TLRs and MTB infection is shown in **Figure 4**.

In addition, TLR4 is an important pattern recognition receptor that is expressed in mononuclear macrophages, dendritic cells and alveolar II-type epithelial cells and plays a crucial role in resistance to MTB infection. In the TLR family, TLR4 is a type I transmembrane protein. However, unlike the TLR2 signal transduction pathway, TLR4 can activate the MyD88-dependent pathway, induce the release of inflammatory cytokines and costimulators, and also activate the MyD88-independent pathway to produce type I interferon (IFN) (Niu et al., 2018). After TLR4 is stimulated by ligands, TLR4 activates transcriptional regulatory factors by connecting and transmitting TLR4 downstream signals through transduction molecules, such as NF-κB, inducing infected cells to secrete inflammatory factors and type I IFN, which ultimately initiates innate and acquired immune responses (Thada et al., 2021). TLR4 was found to be involved in LPS-induced activation of alveolar II-type epithelial cells (AEC II), leading to the large-scale release of TNF-α and IL-6, while single immunoglobin IL-1 receptor-related protein (SI-GIRR) overexpression inhibited TLR4 signal transduction in LPS-triggered AEC II, alleviating the inflammatory response (Alemnew et al., 2021). Recent studies have found that AEC II cells infected with strong MTB strains demonstrate an inhibition trend on TLR2 and TLR4 signaling pathways, and the inflammatory response of cells is significantly stronger than that of weak MTB strains, indicating that AEC II also differ in the immune regulation mechanism of MTB infection with different virulence levels (Krutzik et al., 2003). Overexpression of TLR2 and TLR4 in human cell lines is directly related to MTB infection (Kang et al., 2002). Furthermore, polymorphisms in both TLR2 and TLR4 are possibly associated with increased susceptibility to MTB infection (Lorenz et al., 2000; Rook et al., 2005).

At present, an increasing number of clinical diagnostic studies have confirmed that the gene expression levels of the TLR family are closely related to MTB patients. For example, the TLR distribution in MTB granuloma lesions indicates that TLR1, TLR2, and TLR4 are expressed in both immune cells and nonimmune cells; however, TLR9 is only detectable in immune cells (Martinez-Perez et al., 2020). Interestingly, similar results were found in which the relative mRNA expression levels of TLR2, TLR4, and TLR8 in tuberculosis patients were significantly increased (Davila et al., 2008). Furthermore, in an animal model of TB, TLR8-deficient mice succumb more rapidly to MTB infection, despite efficiently controlling the number of viable bacilli in different organs. Thus, in this model of MTB infection, TLR8 plays a key role in dampening inflammation and tissue damage (Najmi et al., 2010).

Other studies have also confirmed that the expression and activation of TLRs can be used as auxiliary indicators for the diagnosis and differential diagnosis of tuberculosis. For instance, mice lacking the TLR ligand protein MyD88 are more sensitive to MTB infection, and the same phenomenon was found following TLR2 gene deletion. However, whether the TLR signaling pathway resists MTB infection is controversial; for instance, it has been reported that MyD88 rather than TLRs plays a key role in the activation of the macrophage response, and the release of cytokines does not depend on TLRs (Kramnik and Beamer, 2016).

### Molecular mechanism of MTB escaping the host NLR receptor signal

Recently, the role of the cytoplasmic receptor NLR in microbial infection has attracted much attention. The nucleotide oligomerization domain (NOD)-like receptor (NLR) is a kind of innate immune intracellular recognition receptor in the cell cytoplasm that plays a key role in the regulation of innate immunity (Nargis et al., 2016; Alvarez-Jiménez et al., 2018). The interaction between monocyte-derived dendritic cells and MTB was observed under an electron microscope. It was found that MTB could escape from the phagocyte to the cytoplasm depending on early secretory antigen target (ESXA). ESXA is also a cell wall-binding protein with periodic replication from phagocytosed lysosomes to the cytoplasm (Tan et al., 2020). Thus, cytoplasmic pattern recognition receptors are also involved in host innate and acquired immunity against MTB.

The NLR acts as a scaffold protein in the cytoplasm, assembling signaling platforms that trigger the NF-κB and mitogen-activated protein kinase (MAPK) signaling pathways and control inflammatory caspase activation. Muramyl dipeptide (MDP) of MTB recognized by NOD2 can stimulate the release of host inflammatory factors and nitrogen oxides (NO) to play a bactericidal role, and activated NOD2 induces autophagy (Cubillos-Angulo et al., 2021). The MTB MDP is unique in that it has an N-glycolyl modification rather than the N-acetylated modification found in other bacteria (Donovan et al., 2017). Studies have demonstrated that MDP in MTB can promote macrophages through the receptor-interacting protein kinase 2 (PIP2) and interferon regulatory factor 5 (IRF5) pathways, producing type I IFN signaling, such as IFN-α/β, which counteracts the role of the host protective IL-1β and IFN-γ signaling pathways (Sabir et al., 2017). Mice with type I IFN receptor deficiency did not have altered MTB burdens in the lungs, possibly even promoting the reproduction of extrapulmonary bacteria (Wan et al., 2018). Furthermore, the cytoplasmic receptor NLR plays a role in pyroptosis in host cells. Studies have demonstrated that, after MTB activates host cells, NLR is recruited to the inflammasomes of the macromolecular complex, causing host cells to secrete IL-1β, IL-18, and IL-33 and leading to pyroptosis (Qu et al., 2020). In addition, the inflammation caused by MTB infection of host cells is mainly related to NLRP3 in the NLR family (Beckwith et al., 2020). Recent studies have demonstrated that, in human macrophages, MTB relies on ESAT-6 to activate the inflammatory response and release IL-1β, leading to pyroptosis, while in mouse dendritic cells, IL-1β secretion is independent of NLRP3, and no evidence of pyroptosis has been found (Wilson et al., 2015).

### Molecular mechanism of MTB evasion of host CLR receptor signaling

C-type lectin receptors (CLRs) are a superfamily of proteins containing carbon hydrate recognition domains (CRDs). According to subcellular localization, it can be divided into two categories, soluble and membrane-type C-type lectins, and it is a crucial pattern recognition receptor (PRR) in the innate immune system (Zhao et al., 2014). Mycobacterium cord factor TDM (trehalose-6,6’-dimycolate) is a glycosolipid of mycoacid and trehalose that exists in the outer layer of bacterial cells. Studies have confirmed that TDM can bind to macrophage C-type lectin receptor (CLR) to induce changes in downstream pathways (Barkan et al., 2010).

Interestingly, researchers have found that TDM-induced acellular granulomas are very similar to *Mycobacterium tuberculosis*-infected granulomas when TDM-emulsified oil droplets are injected into mice (Behr et al., 2015). The genes responsible for TDM synthesis in MTB include cyclopropane synthase PcaA and CmaA2. If these genes are deleted, the structure of Cladophyll acid changes, and the host’s immune response changes accordingly. Recently, it was found that mutant strains of MTB-PcaA lacking cyclopropane synthase induced a reduced ability of wild-type mouse macrophages to produce proinflammatory cytokines and to form new granulomas (Hayley et al., 2017).

Studies have confirmed that, after the binding of TDMS receptors to CLRs, macrophages are activated through the SYK/CARD9/BCL10 pathway to produce cytokines (Mohlopheni et al., 2010). Further studies have shown that CARD9-deficient mice can initiate an effective adaptive immune response, but the growth of MTB in host cells is not restricted (Matthew Wagener et al., 2018). These findings suggested that MTB can produce an inflammatory level sufficient to promote granulomatous development without stimulating host cell death by fine-tuning mycoacid synthesis, allowing MTB to slowly persist or proliferate, thereby evading the host immune response.

## Molecular mechanism of MTB escaping the host macrophage effect

After being engulfed by macrophages, MTB can block the fusion of phagocytes and lysosomes, thus avoiding lysosomal attack. The phagosome of *Mycobacteri*a cannot be incorporated into the vesicular ATP enzyme; thus, the transmembrane transport of the intimal system cannot be controlled by the energy produced by hydrolyzing ATP (Yu et al., 2018). The specific molecular mechanism by which MTB inhibits phagosome-lysosome fusion is not yet known and needs further study. At present, it is believed that the serine/threonine protein kinases (STPKs) of MTB are effective molecules for inhibiting phagosome-lysosome fusion (Shukla et al., 2014). A recent study showed that MTB containing STPKs could inhibit phagosome-lysosomal fusion, while MTB without STPKs could not locate the lysosome-like structure of host cells (Lee et al., 2018). It is suggested that the mechanism may be related to the regulation of the tricarboxylic acid cycle and the perception of amino acid content by effector molecules containing Ser/Thr kinase through the phosphorylated substrate GarA (Alber, 2009; Pahari et al., 2020).

## Molecular mechanism of MTB escaping host autophagy

Autophagy is a process in which the host is responsible for cell homeostasis and can be induced, which can remove damaged organelles or unnecessary subcellular structures. Autophagy first forms autophagic vesicles with a double-membrane structure. When the inclusion bodies in the cytoplasm fuse with lysosomes, the substances swallowed by autophagy vesicles are digested.

Research has found that MTB can be located in autophagy vesicles in host cells, and lysosomes obtained from phagocytes containing MTB can cause autophagy in host cells by acidification, thus eliminating MTB. After lipopolysaccharide treatment of host cells, the number of MTB in autophagy vesicles increases sharply, which suggests that TLR4 mediates autophagy induction (Mohd et al., 2021; Romagnoli et al., 2018). A further study found that MTB activates macrophage autophagy mainly by intracellular vitamin D receptor and IL-1R (Degiacomi et al., 2017; Strong et al., 2020).

The antigenic target protein ESX-1 of MTB is a multibasement membrane complex that forms a channel on the membrane (Niu et al., 2011). Recent research found that ESX-1 can increase the permeability of phagocytes and activate NLRP3 receptors. When the ESX-1 content decreases, the virulence of MTB also decreases. In addition, MTB can use the host miRNA pathway to coordinate autophagy and reprogram host lipid metabolism to ensure its intracellular survival and retention (Martinez et al., 2016; Mirzaei et al., 2021).

## Summary and prospects

In this review, we focus on the special cell structure of MTB and the research progress of the interaction between MTB and host cell surface pattern recognition receptors (such as CLRs, NLRs, and TLRs), as well as macrophage effector molecules and autophagy. At present, through the study of these signaling pathways, researchers have a certain understanding of the mechanism by which MTB resides in the host but a very limited understanding of the molecular mechanism of escape from host pattern recognition receptor signal transduction. MTB can change host cell immune function through a variety of molecular mechanisms (Bussi and Gutierrez, 2019). In this paper, we reviewed a large number of papers about how the immune system senses and fights MTB and how MTB escapes the immune system. We also introduced the structure of MTB, the interaction of MTB effector proteins and the host and the molecular mechanism of MTB escape from host membrane surface pattern receptors. This study may provide research ideas for the discovery of new anti-tuberculosis drugs and the development of host-oriented treatment strategies.

In the future, we should continue exploring the effector components of MTB interacting with host cells through various effective screening techniques, such as transposon mutation libraries (López-Agudelo et al., 2020) and Crispr-Cas9 mutation libraries (Qiyao et al., 2020). In addition, the protein–protein interactions between MTB and host cells usually involve bacterial pathogenicity and persistence, and these proteins play a potential role in the pathogenesis of tuberculosis. Therefore, further understanding of the MTB-host molecular interaction network will be helpful to understand the molecular mechanisms by which MTB escape the host’s immune response. These studies will help identify new methods and strategies for the prevention and treatment of tuberculosis and lay a foundation for the early eradication of tuberculosis.

## Author Contributions

All authors listed have made a substantial, direct, and intellectual contribution to the work and approved it for publication. Conceptualization, QL and LG; validation, QL and LG; formal analysis, YW and QS; investigation, YW and QS; data curation, QC, XZ and HY; writing—original draft preparation, QL and LG; writing—review and editing, LG; SL and YW; supervision, QL and LG; project administration, LG; funding acquisition, QL and LG.

## Funding

This research was supported by the National Natural Science Foundation of China (No. 81673639) and the Natural Science Foundation of Zhejiang Province (LY20H280002).and Zhejiang Chinese Medical University (No. 2022TS001).

## Conflict of Interest

The authors declare that the research was conducted in the absence of any commercial or financial relationships that could be construed as a potential conflict of interest.

## Publisher’s Note

All claims expressed in this article are solely those of the authors and do not necessarily represent those of their affiliated organizations, or those of the publisher, the editors and the reviewers. Any product that may be evaluated in this article, or claim that may be made by its manufacturer, is not guaranteed or endorsed by the publisher.

## References

[B1] Abrahams K. A. Besra G. S. (2018). Mycobacterial cell wall biosynthesis: A multifaceted antibiotic target. Parasitology 145, 116–133. doi: 10.1017/S0031182016002377 27976597 PMC5964476

[B84] Alber T. (2009). Signaling mechanisms of the mycobacterium tuberculosis receptor Ser/Thr protein kinases. Curr. Opin. Struct. Biol. 19 (6), 650–657. doi: 10.1016/j.sbi.2009.10.017 19914822 PMC2790423

[B56] Alemnew B. Hoff S. T. Abebe T. Abebe M. Aseffa A. Howe R.. (2021). *Ex vivo* mRNA expression of toll-like receptors during latent tuberculosis infection. BMC Immunol. 22 (9). doi: 10.1186/s12865-021-00400-4 PMC7842038 33509080

[B28] Allue-Guardia A. Garcia J. I. Torrelles J. B. (2021). Evolution of drug-resistant mycobacterium tuberculosis strains and their adaptation to the human lung environment. Front. Microbiol. 12, 612675. doi: 10.3389/fmicb.2021.612675 33613483 PMC7889510

[B66] Alvarez-Jiménez V. D. Leyva-Paredes K. García-Martínez M. Vázquez-Flores L. García-Paredes V. G. Campillo-Navarro M.. (2018). Extracellular vesicles released from mycobacterium tuberculosis-infected neutrophils promote macrophage autophagy and decrease intracellular mycobacterial survival. Front. Immunol. 9, 272. doi: 10.3389/fimmu.2018.00272 29520273 PMC5827556

[B10] Arcos J. Sasindran S. J. Fujiwara N. Turner J. Schlesinger L. S. Torrelles J. B. (2011). Human lung hydrolases delineate mycobacterium tuberculosis-macrophage interactions and the capacity to control infection. J. Immunol. 187, 372–381. doi: 10.4049/jimmunol.1100823 21602490 PMC4201034

[B24] Augenstreich J. Briken V. (2020). Host cell targets of released lipid and secreted protein effectors of mycobacterium tuberculosis. Front. Cell Infect. Microbiol. 10, 595029. doi: 10.3389/fcimb.2020.595029 33194845 PMC7644814

[B2] Chakaya J Khan M Ntoumi F Aklillu E Fatima R Mwaba P. (2020). Global tuberculosis report 2020 (Geneva: World Health Organization). Licence: CC BY-NC-SA 3.0 IGO.

[B76] Barkan D. Rao V. George D. Sukenick G. D. Glickman M. S.. (2010). Redundant function of cmaA2 and mmaA2 in mycobacterium tuberculosis cis cyclopropanation of oxygenated mycolates. J. Bacteriol. 192 (14), 3661–3668. doi: 10.1128/JB.00312-10 20472794 PMC2897352

[B4] Barreteau H. Kovac A. Boniface A. Sova M. Gobec S. Blanot D. (2008) Cytoplasmic steps of peptidoglycan biosynthesis. FEMS Microbiol. Rev. 32, 168–207. doi: 10.1111/j.1574-6976.2008.00104.x 18266853

[B73] Beckwith K. S. Beckwith M. S. Ullmann S. Sætra R. S. Kim H. Marstad A.. (2020). Plasma membrane damage causes NLRP3 activation and pyroptosis during mycobacterium tuberculosis infection. Nat. Commun. 11, 2270. doi: 10.1038/s41467-020-16143-6 32385301 PMC7210277

[B77] Behr M. A. Divangahi M. (2015). Freund’s adjuvant, NOD2 and mycobacteria. Curr. Opin. Microbiol. 23, 126–132. doi: 10.1016/j.mib.2014.11.015 25483349

[B13] Benjawan K. Vivek N. Sittiruk R. Namwat W Paemanee A Lulitanond V.. (2015). Comparative proteomics of activated THP-1 cells infected with mycobacterium tuberculosis identifies putative clearance biomarkers for tuberculosis treatment. PloS One 10 (7), e0134168. doi: 10.1371/journal.pon 26214306 PMC4516286

[B3] Brennan P. J. Crick D. C. (2007). The cell-wall core of mycobacterium tuberculosis in the context of drug discovery. Curr. Top. Med. Chem. 7, 475–488. doi: 10.2174/156802607780059763 17346193

[B46] Bulut Y. Michelsen K. S. Hayrapetian L. Naiki Y. Spallek R. Singh M.. (2005). Mycobacterium tuberculosis heat shock proteins use diverse toll-like receptor pathways to activate pro-inflammatory signals. J. Biol. Chem. 280 (22), 20961–20967. doi: 10.1074/jbc.M411379200 15809303

[B93] Bussi C. Gutierrez M. G. (2019). Mycobacterium tuberculosis infection of host cells in space and time 43, 4, 341–361. doi: 10.1093/femsre/fuz006 PMC6606852 30916769

[B8] Canezin P. H. Caleffi-Ferracioli K. R. Scodro R. B. L. Dias Siqueira V. L. Pavan F. R. Esteves Barros I. L.. (2018). Intramacrophage mycobacterium tuberculosis efflux pump gene regulation after rifampicin and verapamil exposure. J. Antimicrob. Chemother. 73, 1770–1776. doi: 10.1093/jac/dky091 29579201

[B30] Cervantes J. L. (2017). MyD88 in mycobacterium tuberculosis infection. Med. Microbiol. Immunol. 206, 187–193. doi: 10.1007/s00430-017-0495-0 28220253

[B68] Cubillos-Angulo J. M. Fernandes C. D. Araújo D. N. Carmo C. A. Arriaga M B. and Andrade B. B. (2021). The influence of single nucleotide polymorphisms of NOD2 or CD14 on the risk of mycobacterium tuberculosis diseases: A systematic review. Syst. Rev. 10, 174. doi: 10.1186/s13643-021-01729-y 34108050 PMC8191055

[B62] Davila S. Hibberd M. L. HariDass R. Wong H. E.E. Sahiratmadja E. Bonnard C.. (2008). Genetic association and expression studies indicate a role of toll-like receptor 8 in pulmonary tuberculosis. PloS Genet. 4, e1000218. doi: 10.1371/journal.pgen.1000218 18927625 PMC2568981

[B89] Degiacomi G. Benjak A. Madacki J. Boldrin F. Provvedi R. Palù G.. (2017). Essentiality of mmpL3 and impact of its silencing on mycobacterium tuberculosis gene expression. Sci. Rep. 7, 43495. doi: 10.1038/srep43495 28240248 PMC5327466

[B69] Donovan M. L. Schultz T. E. Duke T. J. Blumenthal A. (2017). Type I interferons in the pathogenesis of tuberculosis: Molecular drivers and immunological consequences. Front. Immunol. 8, 1633. doi: 10.3389/fimmu.2017.01633 29230217 PMC5711827

[B41] Doyle S. L. O’Neill L. A. (2006). Toll-like receptors: from the discovery of NFkappaB to new insights into transcriptional regulations in innate immunity. Biochem. Pharmacol. 72, 1102–1113. doi: 10.1016/j.bcp.2006.07.010 16930560

[B48] Drage M. G. Pecora N. D. Hise A. G. Febbraio M. Silverstein R. L. Golenbock D. T.. (2009). TLR2 and its coreceptors determine responses of macrophagesand dendritic cells to lipoproteins of mycobacterium tuberculosis. Cell Immunol. 258, 29–37. doi: 10.1016/j.cellimm.2009.03.008 19362712 PMC2730726

[B43] Fiske C. T. Blackman A. Maruri F. Rebeiro P. F. Huaman M. Kator J.. (2019). Increased vitamin d receptor expression from macrophages after stimulation with m. tuberculosis among persons who have recovered from extrapulmonary tuberculosis. BMC Infect. Dis. 19, 366. doi: 10.1186/s12879-019-3958-7 31039752 PMC6492421

[B20] Foot Perkowski E. Miller B. K. McCann J. R. Sullivan J. T. Malik S. Coy Allen I.. (2016). An orphaned mce-associated membrane protein of mycobacterium tuberculosis is a virulence factor that stabilizes mce transporters. Mol. Microbiol. 100 (1), 90–107. doi: 10.1111/mmi.13303 26712165 PMC5028898

[B78] Hayley C. W. DiFazio R. M. Linderman J. J. Flynn J. L. Kirschner D. E. (2017). Identifying mechanisms driving formation of granuloma-associated fibrosis during mycobacterium tuberculosis infection. J. Theor. Biol. , 429: 1–429:17. doi: 10.1016/j.jtbi.2017.06.017 PMC5576548 28642013

[B37] Hossain M. M. Norazmi M. N. (2013). Pattern recognition receptors and cytokines in mycobacterium tuberculosis infection-the doubleedged sword. BioMed. Res. Int. 2013, 179174. doi: 10.1155/2013/179174 24350246 PMC3844256

[B27] Jain N. Kalam H. Singh L. Sharma V. Kedia S. Das P.. (2020). Mesenchymal stem cells offer a drug-tolerant and immune-privileged niche to mycobacterium tuberculosis. Nat. Commun. 11, 3062. doi: 10.1038/s41467-020-16877-3 32546788 PMC7297998

[B14] Ji-Hae P. Dahee S. Keu Eun San K. Lee W. Shin S. J. (2021). Understanding metabolic regulation between host and pathogens: New opportunities for the development of improved therapeutic strategies against mycobacterium infection. Front. Cell Infect. Microbiol. 11, 635335. doi: 10.3389/fcimb.2021.635335 33796480 PMC8007978

[B16] Jing W. Teng J. L. L. Zhao D. Ge P. Li B. Woo P. C. Y. (2016). The ubiquitin ligase TRIM27 functions as a host restriction factor antagonized by mycobacterium tuberculosis PtpA during mycobacterial infection. Sci. Rep. 6, 34827. doi: 10.1038/srep34827 27698396 PMC5048167

[B12] Kallenius G. Correia M. Buteme H. Hamasur B. Svenson S. B. (2016). Lipoarabinomannan, and its related glycolipids, induce divergent and opposing immune responses to mycobacterium tuberculosis depending on structural diversity and experimental variations. Tuberculosis (Edinb) 96, 120–130. doi: 10.1016/j.tube.2015.09.005 26586646

[B6] Kalscheuer R. Palacios A. Anso I. Cifuente J. Anguita J. Jacobs W. R. Jr. (2019). The mycobacterium tuberculosis capsule: A cell structure with key implications in pathogenesis. Biochem. J. 476, 1995–2016. doi: 10.1042/BCJ20190324 31320388 PMC6698057

[B58] Kang T. J. Lee S. B. Chae G. T. (2002). A polymorphism in the toll-like receptor 2 is associated with IL-12 production from monocyte in lepromatous leprosy. Cytokine 20, 56–62. doi: 10.1006/cyto.2002.1982 12445799

[B7] Kanji A. Hasan R. Hasan Z. (2019). Efflux pump as alternate mechanism for drug resistance in mycobacterium tuberculosis. Indian J. Tuberc 66, 20–25. doi: 10.1016/j.ijtb.2018.07.008 30797276

[B39] Kawai T. Akira S. (2006). TLR signaling. Cell Death Differ. 13, 816–25. doi: 10.1038/sj.cdd.4401850 16410796

[B32] Kawai T. Akira S. (2011). Toll-like receptors and their crosstalk with other innate receptors in infection and immunity. Immun 34, 637–50. 36. doi: 10.1016/j.immuni.2011.05.006 21616434

[B35] Kay E. Scotland R. S. Whiteford J. R. (2013). Toll-like receptors: role in inflammation and therapeutic potential. Biofactors 40 (3), 284–94. doi: 10.1002/biof.1156 24375529

[B49] Kleinnijenhuis J. Joosten L. A. van de Veerdonk F. L. Savage N. van Crevel R. Jan Kullberg B.. (2009). Transcriptional and inflammasome mediated pathways for the induction of IL-1beta production by mycobacterium tuberculosis. Eur. J. Immunol. 39 (7), 1914–1922. doi: 10.1002/eji.200839115 19544485

[B9] Kleinnijenhuis J. Oosting M. Joosten L. A. Netea M. G. Van Crevel R. (2011). Innate immune recognition of mycobacter-ium tuberculosis. Clin. Dev. Immunol. 405310, 2011. doi: 10.1155/2011/405310 PMC3095423 21603213

[B51] Konowich J. Gopalakrishnan A. Dietzold Verma S. Bhatt K. Rafi W.. (2017). Divergent functions of toll-like receptor 2 on hematopoietic and non-hematopoietic cells during chronic mycobacterium tuberculosis infection. J. Immunol. 198 (2), 741–748. doi: 10.4049/jimmunol.1601651 27920273 PMC5224966

[B31] Krakauer T.. (2019). Inflammasomes, autophagy, and cell death: The trinity of innate host defense against intracellular bacteria. Mediators Inflammation 2471215, 2019. doi: 10.1155/2019/2471215 PMC6341260 30728749

[B64] Kramnik I. Beamer G. (2016). Mouse models of human TB pathology: roles in the analysis of necrosis and the development of host-directed therapies. Semin. Immunopathol. 38, 221–237. doi: 10.1007/s00281-015-0538-9 26542392 PMC4779126

[B57] Krutzik S. R. Ochoa M. T. Sieling P. A. Uematsu S. Ng Y. W. Legaspi A.. (2003). Activation and regulation of toll-like receptors 2 and 1 in human leprosy. Nat. Med. 9, 525–532. doi: 10.1038/nm864 12692544

[B34] Kumar V. (2018). Toll-like receptors in immunity and inflammatory diseases: Past, present, and future. Int. Immunopharmacol 59, 391–412. doi: 10.1016/j.intimp.2018.03.002 29730580 PMC7106078

[B83] Lee H.-J. Ko H.-J. Song D.-K. Jung Y.J. (2018). Lysophosphatidylcholine promotes phagosome maturation and regulates inflammatory mediator production through the protein kinase a–phosphatidylinositol 3 Kinase–p38 mitogen-activated protein kinase signaling pathway during mycobacterium tuberculosis infection in mouse macrophages. Front. Immunol. 9, 920. doi: 10.3389/fimmu.2018.00920 29755479 PMC5934435

[B22] Li J. Chai Q.-Y. Zhang Y. Li B.-X. Wang J. Qiu X.-B.. (2015). Mycobacterium tuberculosis Mce3E suppresses host innate immune responses by targeting ERK1/2 signaling. J. Immunol. 194 (8), 3756–3767. doi: 10.4049/jimmunol.1402679 25780035

[B42] Liu S. Jia H. Hou T. X. Guo X. Zhang G. (2018). Recombinant Mtb9.8 of mycobacterium bovis stimulates TNF-α and IL-1β secretion by RAW264.7 macrophages through activation of NF-κB pathway *via* TLR2. Sci. Rep. 8 (1), 1928. doi: 10.1038/s41598-018-20433-x. 29386556 PMC5792469

[B40] Liu P. T. Krutzik S. R. Modlin R. L. (2007). Therapeutic implications of the TLR and VDR partnership. Trends Mol. Med. 13, 117–124. doi: 10.1016/j.molmed.2007.01.006 17276732

[B36] Liu Y. Yin H. Zhao M. Lu Q.. (2013). TLR2 and TLR4 in autoimmune diseases: a comprehensive review. Clin. Rev. Allergy Immunol. 47 (2), 136–47. 46. doi: 10.1007/s12016-013-8402-y. 24352680

[B44] Liu L. Zhai K. Chen Y. Chen X. Wang G. Wu L.. (2021). Effect and mechanism of mycobacterium tuberculosis lipoprotein LpqH in NLRP3 inflammasome activation in mouse ana-1 macrophage. BioMed. Res. Int. 2021, 8239135. doi: 10.1155/2021/8239135 33490276 PMC7803426

[B94] López-Agudelo V. A. Tom A. Laing E. Wu H. Baena A. Barrera L. F.. (2020). A systematic evaluation of mycobacterium tuberculosis genome-scale metabolic networks. PloS Comput. Biol. 16 (6), e1007533. doi: 10.1371/journal.pcbi.1007533 32542021 PMC7316355

[B59] Lorenz E. Mira J. P. Cornish K. L. Arbour N. C. Schwartz D. A. (2000). A novel polymorphism in the toll-like receptor 2 gene and its potential association with staphylococcal infection. Infect. Immun. 68, 6398–6401. doi: 10.1128/IAI.68.11.6398-6401.2000 11035751 PMC97725

[B5] Marrakchi H. Laneelle M. A. Daffe M. (2014). Mycolic acids: structures, biosynthesis, and beyond. Chem. Biol. 21, 67–85. doi: 10.1016/j.chembiol.2013.11.011 24374164

[B91] Martinez N. Ketheesan N. West K. Vallerskog T. Kornfeld H. (2016). Impaired recognition of mycobacterium tuberculosis by alveolar macrophages from diabetic mice. J. Infect. Dis. 214, 1629–1637. doi: 10.1093/infdis/jiw436 27630197 PMC5144731

[B61] Martinez-Perez A. Igea A. Estevez Ferreira C. M. Torrado E. Gil Castro A.. (2020). Changes in the immune phenotype and gene expression profile driven by a novel tuberculosis nanovaccine: Short and long-term post-immunization. Front. Immunol. 11, 589863. doi: 10.3389/fimmu.2020.589863 33584654 PMC7876410

[B80] Matthew Wagener J. Hoving C. Ndlovu H. Marakalala M. J.. (2018). Dectin-1-Syk-CARD9 signaling pathway in TB immunity. Front. Immunol. 9, 225. doi: 10.3389/fimmu.2018.00225 29487599 PMC5816931

[B53] Mehta P. Ray A. Mazumder S. (2021). TLRs in mycobacterial pathogenesis: Black and white or shades of Gray. Curr. Microbiol 78 (6), 2183–2193. doi: 10.1007/s00284-021-02488-8 33844035

[B52] Mercedes Romero M. Ignacio Basile J. López B. Ritacco V. Barrera L. del Carmen Sasiain M.. (2014). Outbreaks of mycobacterium tuberculosis MDR strains differentially induce neutrophil respiratory burst involving lipid rafts, p38 MAPK and syk. BMC Infect. Dis. 14, 262. doi: 10.1186/1471-2334-14-262 24886274 PMC4049492

[B25] Mika J. Zychowska M. Popiolek-Barczyk K. Rojewska E. Przewlocka B. (2013). Importance of glial activation in neuropathic pain. Eur. J. Pharmacol. 716, 106–119. doi: 10.1016/j.ejphar.2013.01.072 23500198

[B92] Mirzaei R. Babakhani S. Ajorloo P. Heidari Ahmadi R. Hosseini-Fard S. R. Keyvani H.. (2021). The emerging role of exosomal miRNAs as a diagnostic and therapeutic biomarker in mycobacterium tuberculosis infection. Mol. Med. 27, 34. doi: 10.1186/s10020-021-00296-1 33794771 PMC8017856

[B17] Mohd S. Neha Q. Javaid Ahmad S. Kumar Singh A. Bishai W. R. Ehtesham N. Z.. (2021). Post translational modifications in tuberculosis: ubiquitination paradox. Autophagy 17 (3), 814–817. doi: 10.1080/15548627.2020.1850009 33190592 PMC8032244

[B86] Mohd S. Neha Q. Neha S. Singh J. Sheikh J. A. Khubaib M.. (,2021). Mycobacterium tuberculosis RipA dampens TLR4-mediated host protective response using a multi-pronged approach involving autophagy, apoptosis, metabolic repurposing, and immune modulation. Front. Immunol. 12, 636644. doi: 10.3389/fimmu.2021.636644 33746976 PMC7969667

[B79] Mohlopheni J. M. Graham L. M. Brown G. D. (2010). The role of Syk/CARD9-coupled c-type lectin receptors in immunity to mycobacterium tuberculosis infections. Clin. Dev. Immunol. 2010, 567571. doi: 10.1155/2010/567571 21274433 PMC3025359

[B63] Najmi N. Kaur G. Sharma S. K. Mehra N. K. (2010). Human toll-like receptor 4 polymorphisms TLR4 Asp299Gly and Thr399Ile influence susceptibility and severity of pulmonary tuberculosis in the Asian Indian population. Tissue Antigens 76, 102–109. doi: 10.1111/j.1399-0039.2010.01481.x 20403143

[B65] Nargis K. Pahari S. Vidyarthi A. Aqdas M. Agrewala J. N.. (2016). NOD-2 and TLR-4 signaling reinforces the efficacy of dendritic cells and reduces the dose of TB drugs against mycobacterium tuberculosis. J. Innate Immun. 8 (3), 228–242. doi: 10.1159/000439591 26613532 PMC6738777

[B38] Nguyen H. Gazy N. Venketaraman V. (2020). A role of intracellular toll-like receptors (3, 7, and 9) in response to mycobacterium tuberculosis and Co-infection with HIV. Int. J. Mol. Sci. 21 (17), 6148. doi: 10.3390/ijms21176148 PMC7503332 32858917

[B90] Niu H. Hu L. Li Q. Da Z. Wang B. Tang K.. (2011). Construction and evaluation of a multistage mycobacterium tuberculosis subunit vaccine candidate Mtb10.4-HspX. Vaccine 29, 9451–9458. doi: 10.1016/j.vaccine.2011.10.032 22024175

[B54] Niu W. Sun B. Li M. Cui J. Huang J. Zhang L.. (2018). TLR-4/microRNA-125a/NF-κB signaling modulates the immune response to mycobacterium tuberculosis infection. Cell Cycle 17 (15), 1931–1945. doi: 10.1080/15384101.2018.1509636 30153074 PMC6152532

[B85] Pahari S. Negi S. Aqdas M. Arnett E. Schlesinger L. S. Agrewala J. N.. (2020). Induction of autophagy through CLEC4E in combination with TLR4: an innovative strategy to restrict the survival of mycobacterium tuberculosis. Autophagy 16 (6), 1021–1043. doi: 10.1080/15548627.2019.1658436 31462144 PMC7469444

[B29] Pathak L. Das B. (2020). Initiation of post-primary tuberculosis of the lungs: Exploring the secret role of bone marrow derived stem cells. Front. Immunol. 11, 594572. doi: 10.3389/fimmu.2020.594572 33584661 PMC7873989

[B45] Picard C. Puel A. Bonnet M. Ku C.L. Bustamante J. Yang K.. (2003). Pyogenic bacterial infections in humans with IRAK-4 deficiency. Science 299 (5615), 2076–2079. doi: 10.1126/science.1081902 12637671

[B95] Qiyao C. Lin W. Cui Hua L. Ge B.. (2020). New insights into the evasion of host innate immunity by mycobacterium tuberculosis. Cell Mol. Immunol. 17 (9), 901–913. doi: 10.1038/s41423-020-0502-z 32728204 PMC7608469

[B72] Qu Z. Zhou J. Zhou Y. Xie Y. Jiang Y. Wu J.. (2020). Mycobacterial EST12 activates a RACK1–NLRP3–gasdermin d pyroptosis–IL-1β immune pathway. Sci. Adv. 6 (43), eaba4733. doi: 10.1126/sciadv.aba4733 33097533 PMC7608829

[B11] Richa M. Sakshi K. Nitish M. Bandyopadhyay P. Mehta M. Munshi M.. (2019). Targeting redox heterogeneity to counteract drug tolerance in replicating mycobacterium tuberculosis. Sci. Transl. Med. 11 (518), eaaw6635. doi: 10.1126/scitranslmed.aaw6635 31723039 PMC7212044

[B87] Romagnoli A. Petruccioli E. Palucci I. Camassa S. Carata E. Petrone L.. (2018). Clinical isolates of the modern mycobacterium tuberculosis lineage 4 evade host defense in human macrophages through eluding IL-1β-induced autophagy. Cell Death Dis. 9 (6), 624. doi: 10.1038/s41419-018-0640-8 29795378 PMC5967325

[B60] Rook G. A. Dheda K. Zumla A. (2005). Opinion: immune responses to tuberculosisin developing countries: Implications for new vaccines. Nat. Rev. Immunol. 5, 661–667. doi: 10.1038/nri1666 16056257

[B70] Sabir N. Hussain T. Shah S. Z. A. Zhao D. Zhou X.. (2017). IFN-beta: A contentious player in host-pathogen interaction in tuberculosis. Int. J. Mol. Sci. 18 (12), 2725. doi: 10.3390/ijms18122725. PMC5751326 29258190

[B82] Shukla S. Richardson E. T. Athman J. J. Shi L. Wearsch P. A. McDonald D.. (2014). Mycobacterium tuberculosis lipoprotein LprG binds lipoarabinomannan and determines its cell envelope localization to control phagolysosomal fusion. PloS Pathog. 10 (10), e1004471. doi: 10.1371/journal.ppat.1004471 25356793 PMC4214796

[B21] Singh P. Katoch V. M. Mohanty K. K. Chauhan D. S.. (2016). Analysis of expression profile of mce operon genes (mce1, mce2, mce3 operon) in different mycobacterium tuberculosis isolates at different growth phases. Indian J. Med. Res. 143 (4), 487–494. doi: 10.4103/0971-5916.184305 27377506 PMC4928556

[B47] Singh B. Saqib M. Chakraborty A. Bhaskarl S.. (2019). Lipoarabinomannan from mycobacterium indicus pranii shows immunostimulatory activity and induces autophagy in macrophages. PloS One 14 (10), e0224239. doi: 10.1371/journal.pone.0224239 31648257 PMC6812838

[B88] Strong E. J. Jurcic Smith K. L. Saini N. K. Ng T. W. Porcelli S. A. Lee S.. (2020). Identification of autophagy-inhibiting factors of mycobacterium tuberculosis by high-throughput loss-of-Function screening. Infect. Immun. 88 (12), e00269–e00220. doi: 10.1128/IAI.00269-20 32989037 PMC7671894

[B33] Takeuchi O. Akira S.. (2010). Pattern recognition receptors and inflammation. Cell 140, 805–820. doi: 10.1016/j.cell.2010.01.022 20303872

[B67] Tan H. K. Fan S. J. Xu Y. C. Zhou J. J. Chen Y. Z. Xie T. A.. (2020). The clinical diagnostic value of xpert MTB/RIF for the detection of mycobacterium tuberculosis in gastric aspirates. Biosci. Rep. 40. doi: 10.1042/BSR20200138 PMC7313445 32543657

[B55] Thada S. Horvath G. L. Muller M. M. Dittrich N. Conrad M. L. Sur S.. (2021). Interaction of TLR4 and TLR8 in the innate immune response against mycobacterium tuberculosis. Int. J. Mol. Sci. 22 (4), 1560. doi: 10.3390/ijms22041560 33557133 PMC7913854

[B26] Tuladhar S. Kanneganti T. D. (2020). NLRP12 in innate immunity and inflammation. Mol. Aspects Med. 76, 100887. doi: 10.1016/j.mam.2020.100887 32838963 PMC9375713

[B18] Valérie P. Bach H. Av-Gay Y. (2014). Mycobacterium tuberculosis promotes anti-apoptotic activity of the macrophage by PtpA protein-dependent dephosphorylation of host GSK3α. J. Biol. Chem. 289 (42), 29376–29385. doi: 10.1074/jbc.M114.582502 25187516 PMC4200286

[B19] Wang J. Ge P. Qiang L. Tian F. Zhao D. Chai Q.. (2017). The mycobacterial phosphatase PtpA regulates the expression of host genes and promotes cell proliferation. Nat. Commun. 8 (1), 244. doi: 10.1038/s41467-017-00279-z 28811474 PMC5557760

[B71] Wan M. Zhou Y. Zhu Y. (2018). Subversion of macrophage functions by bacterial protein toxins and effectors. Curr. Issues Mol. Biol. 25, 61–80. doi: 10.21775/cimb.025.061 28875940

[B23] Wei S. Wang D. Li H. Bi L. Deng J. Zhu G.. (2019). Fatty acylCoA synthetase FadD13 regulates proinflammatory cytokine secretion dependent on the NF-κB signalling pathway by binding to eEF1A1. Cell Microbiol. 21 (12), e13090. doi: 10.1111/cmi.13090 31364251 PMC6899955

[B74] Wilson G. J. Marakalala M. J. Hoving J. C. van Laarhoven A. Drummond R. A. Kerscher B.. (2015). The c-type lectin receptor Clecsf8/Clec4D is a key component of anti-mycobacterial immunity. Cell Host Microbe 17 (2), 252–259. doi: 10.1016/j.chom.2015.01.004 25674984 PMC4334100

[B15] Wong K. W. (2017). The role of ESX-1 in mycobacterium tuberculosis. Pathogenesis Microbiol. Spectr. 5 (3). doi: 10.1128/microbiolspec.TBTB2-0001-2015. PMC11687508 28513416

[B50] Yang Q. Liao M. Wang W. Zhang M. Chen Q. Guo J.. (2019). CD157 confers host resistance to mycobacterium tuberculosis *via* TLR2-CD157-PKCzeta-Induced reactive oxygen species production. mBio 10 (4), e01949–e01919. doi: 10.1128/mBio.01949-19 31455656 PMC6712401

[B81] Yu H. Lupoli T. J. Kovach A. Meng X Zhao G. Nathan C. F.. (2018). ATP hydrolysis-coupled peptide translocation mechanism of mycobacterium tuberculosis ClpB. Proc. Natl. Acad. Sci. U S A 115 (41), E9560–E9569. doi: 10.1073/pnas.1810648115 30257943 PMC6187150

[B75] Zhao X.-Q. Zhu L.-L. Chang Q. Jiang C. You Y. Luo T.. (2014). C-type lectin receptor dectin-3 mediates trehalose 6,6′-dimycolate (TDM)-induced mincle expression through CARD9/Bcl10/MALT1-dependent NF-κB activation. J. Biol. Chem. 289 (43), 30052–30062. doi: 10.1074/jbc.M114.588574 25202022 PMC4208012

